# Catatonia in adolescence: an approach to genetic disorders, autism spectrum and delay of diagnosis

**DOI:** 10.1192/j.eurpsy.2023.1545

**Published:** 2023-07-19

**Authors:** N. De Sousa Figueiredo

**Affiliations:** Hospital de Navarra, Pamplona

## Abstract

**Introduction:**

Catatonia is a treatable but often undiagnosed condition in children and adolescents. The majority of Pediatric catatonia cases occur at a puberal ages. It is associated with neurodevelopmental disorders. In these cases the diagnosis can be more difficult due to the overlap of symptoms.

**Objectives:**

Report the case of a 11- year - old girl who developed catatonia. She had a previous psychiatry history of intellectual disability, delayed speech and motor slowness. She had a positive Lorazepam challenge test with resolution of the most catatonic symptoms. More studies were completed and according to the clinical history the diagnosis of autistic spectrum disorder was made.

Genetic test revealed a Phelan Mc Dermid Syndrom.

**Methods:**

A year 11-year-old girl presented to the pediatric emergency department with a 2- days history of worsening anxiety and rigidity, seeming lost and distant. The previous 4 months there was a history of progressive functional and social decline. Her speech was minimal and she required assistance with dressing and feeding. She displayed stereotypias and mannerism. All medical studies were unremarkable. A Lorazepam challenge test (2 mg IV) showed evidence of response. She recovered from catatonia and the basal situation was studied. She was diagnosed of autistic espectrum disorder attending the psychiatric and medical history. Apart from other medical studies, a genetic test showed a mutation in a gene called SHANK 3 according to a Phelan Mc Dermid Syndrom.

**Results:**

Pediatric catatonia is associated with neurodevelopmental disorders such as autistic spectrum disorders. There is not always a clear identifiable cause and it is necessary to rule out possible organic causes of pediatric catatonia. The treatment is similar to adults. It is essential to do a complete medical and psychiatric history to an accurate diagnosis such as an autistic spectrum disorder. Genetic testing must be included. In this case, genetics showed a Phelan Mc Dermid Syndrom with a delayed diagnosis. This disorder can cause a wide range of symptoms varying in severity. These symptoms could include global developmental disorders, intellectual disability, delayed speech, autistic spectrum disorders and minor dysmorphic features.

**Conclusions:**

It is crutial to emphasize the high incidence of catatonic symptoms in individuals with Phelan Mc Dermid Syndrome as catatonia often goes unrecognized or undertreated in individuals with developmental disabilities. Significant cognitive and behavioral regression beyond a baseline level of disability has been reported. This case also highlights the relevance of genetic testing in the work of individuals with intellectual disabilities and acute psychiatric illness or regression. Symptoms indicative of catatonia may occur in context of infections, hormonal status and stressful life events. Treatment is centered on the symptoms.

**Disclosure of Interest:**

None Declared

